# Formation of Li_2_CO_3_ Nanostructures for Lithium-Ion Battery Anode Application by Nanotransfer Printing

**DOI:** 10.3390/ma14071585

**Published:** 2021-03-24

**Authors:** Tae Wan Park, Young Lim Kang, Sang Hyeon Lee, Gu Won No, Eun-Soo Park, Chan Park, Junghoon Lee, Woon Ik Park

**Affiliations:** 1 Electronic Convergence Materials Division, Korea Institute of Ceramic Engineering & Technology (KICET), Jinju 52851, Korea; twpark0125@gmail.com; 2 Department of Materials Science and Engineering, Pukyong National University (PKNU), Busan 48513, Korea; dudfla0914@gmail.com (Y.L.K.); shlee5740@gmail.com (S.H.L.); chanpark@pknu.ac.kr (C.P.); 3 Research and Development Center, Eloi Materials Lab (EML) Co. Ltd., Suwon 16229, Korea; nogu0218@naver.com (G.W.N.); eloiml.korea@gmail.com (E.-S.P.); 4 Department of Metallurgical Engineering, Pukyong National University (PKNU), Busan 48513, Korea

**Keywords:** nanotransfer printing, nanostructure, SPS process, lithium-ion battery, Li_2_CO_3_

## Abstract

Various high-performance anode and cathode materials, such as lithium carbonate, lithium titanate, cobalt oxides, silicon, graphite, germanium, and tin, have been widely investigated in an effort to enhance the energy density storage properties of lithium-ion batteries (LIBs). However, the structural manipulation of anode materials to improve the battery performance remains a challenging issue. In LIBs, optimization of the anode material is a key technology affecting not only the power density but also the lifetime of the device. Here, we introduce a novel method by which to obtain nanostructures for LIB anode application on various surfaces via nanotransfer printing (nTP) process. We used a spark plasma sintering (SPS) process to fabricate a sputter target made of Li_2_CO_3_, which is used as an anode material for LIBs. Using the nTP process, various Li_2_CO_3_ nanoscale patterns, such as line, wave, and dot patterns on a SiO_2_/Si substrate, were successfully obtained. Furthermore, we show highly ordered Li_2_CO_3_ nanostructures on a variety of substrates, such as Al, Al_2_O_3_, flexible PET, and 2-Hydroxylethyl Methacrylate (HEMA) contact lens substrates. It is expected that the approach demonstrated here can provide new pathway to generate many other designable structures of various LIB anode materials.

## 1. Introduction

The development of various energy devices has attracted much attention from those who study energy harvesters [1,2,3,4], fuel cells [5,6], photovoltaics [7,8,9], and batteries [10,11] due to the continuous increase in global energy demand. Among these devices, lithium-ion batteries (LIBs) are highly applicable to various future energy device systems, such as large-capacity electrochemical energy storage (ESS) systems [12,13] and electric vehicles (EVs) [14,15,16,17]. Specifically, LIBs have been significantly considered as an important power source for energy systems given their explosive power. For these reasons, many research groups have consistently studied and reported engineering anode and cathode materials for LIB applications [18,19].

However, there are several critical challenges to be resolved before pattern formation of various electrode structures can be effectively achieved, because the lifetime and power density of LIBs depend strongly on the structure of the electrodes. For example, Fe_2_O_3_ multi-shelled hollow sphere LIB anodes show a significantly high, stable capacity of 1702 mA h g^−1^ with a high energy density level compared to the theoretical value of Fe_2_O_3_ (~1000 mA h g^−1^) [20]. The three-dimensional (3D) porous silicon (Si) anodes used in LIBs also show a high reversible capacity of ~1,730 mA h g^−1^ after 200 cycles, which is superior to planar Si structures [21]. These reports assume that not only the crystal structure but also the physical shape (or morphology) of the anode materials, such as Si, hematite (Fe_2_O_3_), and LMO (Li*M*O_2_, *M* = Co, Ni, Mn) can significantly affect the performance of LIBs, implying that control of anode nanostructures is necessary. As one of the strategies, nanoscale pattern formation of lithium carbonate (Li_2_CO_3_) on the LIB anode such as graphite can provide good passivation and may also promote rich solid electrolyte interphase (SEI) at interface of anode and electrolyte [22,23,24,25].

For the past several decades, in order to generate well-defined active nanostructures, various lithography methods have been extensively developed, such as photolithography [26,27], nanoimprint lithography (NIL) [28,29], extreme ultraviolet (EUV) lithography [30,31], directed self-assembly (DSA) of block copolymer (BCP) [32,33,34,35], and nanotransfer printing (nTP) [36,37,38,39]. Among these useful lithography methods, nTP has received considerable attention due to its high resolutions, simple process, and good cost-effectiveness. The nTP approach can be a more useful methodology when combined with other lithography techniques. In addition, the nTP process can also be employed to obtain complex 2D and 3D pattern geometries with a large surface area on the various surfaces, including flat, curved, and flexible substrates [40,41,42].

In this study, we introduce a facile and useful nTP technique for effectively determining the nanostructures of the LIB anode materials on various substrates, such as metal, oxide, and polymer types. First, a high-density Li_2_CO_3_ sputter target, which can be utilized with a sputtering system during the spark plasma sintering (SPS) process, was fabricated. Subsequently, pattern formation of various Li_2_CO_3_ nanostructures, such as line, wave, and dot structures, were created by the nTP process. The transfer-printed functional nanostructures were systematically observed by a field emission scanning electron microscope (FE-SEM). In addition, the effective formation of Li_2_CO_3_ nanopatterns on diverse material surfaces, in this case aluminum (Al), aluminum oxide (Al_2_O_3_), flexible polyethylene terephthalate (PET), and 2-Hydroxylethyl Methacrylate (HEMA) contact lens substrates, was demonstrated.

## 2. Materials and Methods

### 2.1. Fabrication of High-Density Li_2_CO_3_ Sputter Target

**Figure 1:**
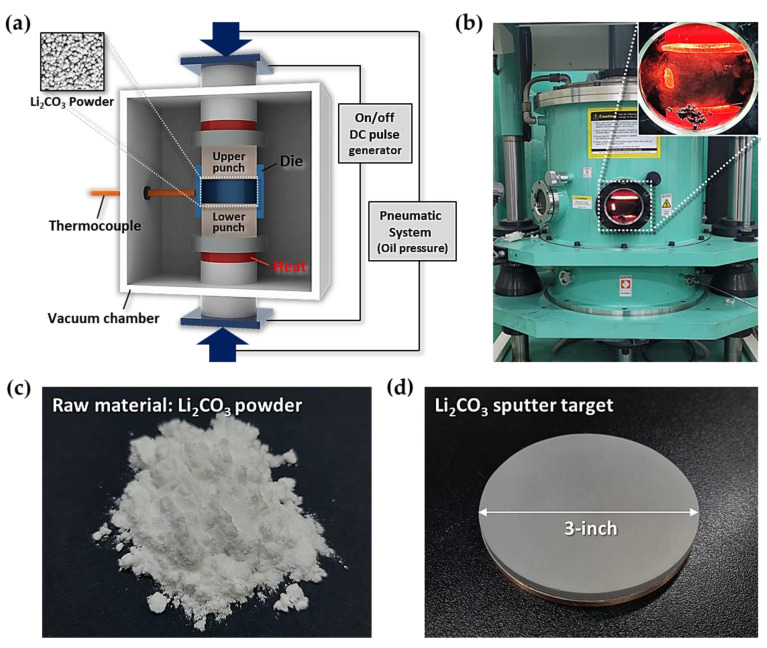
Fabrication of a Li_2_CO_3_ sputter target using a Spark Plasma Sintering (SPS) system. (**a**) Schematic image of the SPS system. (**b**) Photographic image of the working SPS system for the fabrication of the Li_2_CO_3_ sputter target. (**c**) Li_2_CO_3_ raw material (powder). (**d**) Three-inch Li_2_CO_3_ sputter target fabricated by the SPS process.

To obtain Li_2_CO_3_ nanostructures, the fabrication of a Li_2_CO_3_ sputter target is required. We used a SPS system (SPS-3.20MK-V, SPS Syntex Inc, Sojitz Corporation, Tokyo, Japan) to fabricate the sputter target used here from Li_2_CO_3_ powder (99.0%, Junsei Chemical Co. Ltd., Tokyo, Japan). SPS is an optimized rapid sintering method capable of effectively inducing the compaction of ceramic and metal powders at a relatively low temperature for a short time. Figure 1a,b shows schematic and photographic images of the SPS system, respectively. The raw material used for the fabrication of the sputter target is lithium carbonate powder (99.0% Junsei Chemical Co. Ltd., Tokyo, Japan), as shown in Figure 1c. After loading between the upper and lower punches, the Li_2_CO_3_ powder was pressed at 17 MPa of pressure and then sintered at 620 °C for 10 min. The process temperature was determined by controlling the pulsed direct current (4500 A and 4 V). We used a graphite mold (ISO 85) as a die. During the SPS process, the inside of the main chamber was maintained at a 2 × 10^−2^ Torr. After the sintering process, we confirmed the densification of the Li_2_CO_3_ target. A Cu plate was attached onto the backside of the sintered Li_2_CO_3_ body for efficient power injection and rapid cooling when depositing the Li_2_CO_3_ on the substrate. In order to prevent the cracking of the sputter target during the sputtering process, we used Ag paste when bonding the sintered body and the Cu backplate.

### 2.2. Procedure for the Formation of Li_2_CO_3_ Nanostructures via Nanotransfer Printing

For the formation of well-ordered Li_2_CO_3_ nanostructures on the various surfaces used here, we employed the nTP process, which consists of the two steps of replication and printing. In the first step, the replica pattern was formed by a duplication process based on soft polymeric materials. A surface-patterned Si master mold (line width: 250 nm, line space: 250 nm, depth: 250 nm) was prepared by the conventional semiconducting processes of KrF photolithography (NSR-S203B, Nikon, Tokyo, Japan) and dry etching (gas: CF_4_, plasma source power = 250 W, working pressure = 7 mTorr). Before the coating of poly (methyl methacrylate) (PMMA) replica material, the surface of the Si mold was modified with a hydroxyl-terminated poly (dimethylsiloxane) (PDMS) brush at 150 °C for easy separation of the PMMA replica from the Si master mold. PDMS with a molecular weight (MW) of 5 kg/mol (Polymer Source Inc., Dorval, QC, Canada) and PMMA with an MW of 100 kg/mol (Sigma Aldrich Inc., St. Louis, MO, USA) were used. The PDMS was spin-coated after dissolving in toluene solution (2.5 wt.%). To coat the replica material, PMMA powder was dissolved in a binary solvent of toluene and acetone (mixing ratio of two solvents, *V*_tol_/*V*_ace_ = 1) to yield a 4.5 wt.% solution. Toluene (99.5%) and acetone (99.5%) were purchased from Junsei Chemical Co. Ltd., Tokyo, Japan. All spin-coating processes were conducted for 23 seconds at a speed of 5000 rpm. After the PMMA coating step, the PMMA replica pattern was obtained by detaching the spin-coated PMMA thin film from the Si mold using an adhesive polyimide (PI) film (3M Inc., Saint Paul, MN, USA) with a thickness of 150 µm, showing an inverse pattern for the structure of the master mold. In the second step, Li_2_CO_3_ nanopatterns were obtained by transfer printing. The Li_2_CO_3_ active material was deposited on the surface of the PMMA replica pattern by physical vapor deposition (PVD) based on the sputter target. To form Li_2_CO_3_ material selectively only on the protruding part of the replica pattern, a tilt-deposition process was utilized by setting the angle to 50° between the sputtering target and the replica pattern. During the deposition process, high-purity Ar gas (99.99%) was consistently injected into the main chamber. The Li_2_CO_3_ material with a thickness of 15 nm was deposited by means of RF (radio frequency) sputtering at a working pressure of 2 × 10^−5^ Torr with a power level of 100 W for 300 s. To transfer the deposited material onto the target substrate, contact printing was utilized with a heat-rolling-press system (LAMIART-470 LSI, GMP Corp, Korea), as previously reported by the authors [43]. In this system, two rolls that provide both uniform heat and pressure with soft silicone rubber were placed on the top and bottom. During the transfer printing process, the roll speed was held constant at 500 mm/min. When the Li_2_CO_3_ material and the target substrate were passed between the heated rolls, they effectively applied uniform heat and pressure to weaken the adhesion of the adhesive layer of PI film, resulting in the transfer of the PMMA replica and Li_2_CO_3_ material to the target substrate. The PMMA layer was eliminated using an organic solvent (toluene and/or acetone) after the printing process. PMMA residue was perfectly removed by an additional process of O_2_ plasma etching (etching time = 36 s, gas flow rate = 30 s.c.c.m., working pressure = 15 mTorr, and plasma source power = 60 W) with a reactive ion etching (RIE) system. After the selective removal of the PMMA, Li_2_CO_3_ nanostructures were obtained on the target substrates.

## 3. Results and Discussion

To improve the working performance of LIBs based on a recycling strategy, we suggest an advanced nanopatterning method for the raw material of the anode. More specifically, Li_2_CO_3_, an anode material for LIBs, was fabricated as a sputtering target and applied to the pattern transfer printing process. Then, the transfer-printed Li_2_CO_3_ structures were analyzed in detail.

**Figure 2:**
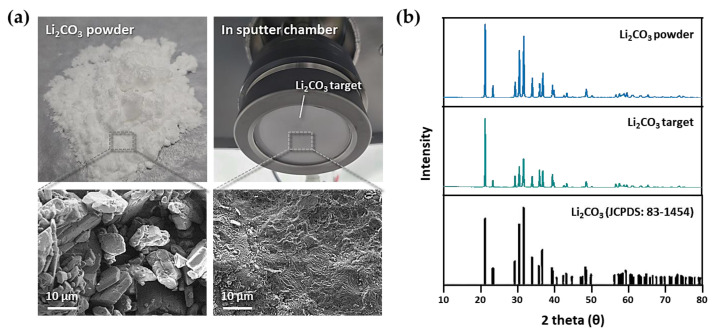
Analysis of the Li_2_CO_3_ powder and Li_2_CO_3_ sputter target. (**a**) SEM images of the surface microstructures of the Li_2_CO_3_ powder and the Li_2_CO_3_ sputter target. (**b**) XRD patterns of the Li_2_CO_3_ powder and Li_2_CO_3_ sputter target.

First, we fabricated a three-inch Li_2_CO_3_ sputter target using the SPS system (Figure 1d). Figure 2a shows the Li_2_CO_3_ raw material (powder) and the Li_2_CO_3_ sputter target from the powder. The lower SEM images are magnified surface microstructures of the upper images. The target with higher density, to which high pressure and a high temperature were applied through the SPS system, was formed compared to the initial powder. Figure 2b shows the X-ray diffraction (XRD) patterns of the powder and the target. All diffraction pattern peaks were well indexed based on lithium carbonate (Joint Committee in Powder Diffraction Standards (JCPDS) No. 83-1454). No peak shift was observed in any of the sintered specimen or the raw powder. This result indicates that the dense crystalline Li_2_CO_3_, which can be used as a sputtering target, is created by the SPS of the Li_2_CO_3_ powder.

**Figure 3:**
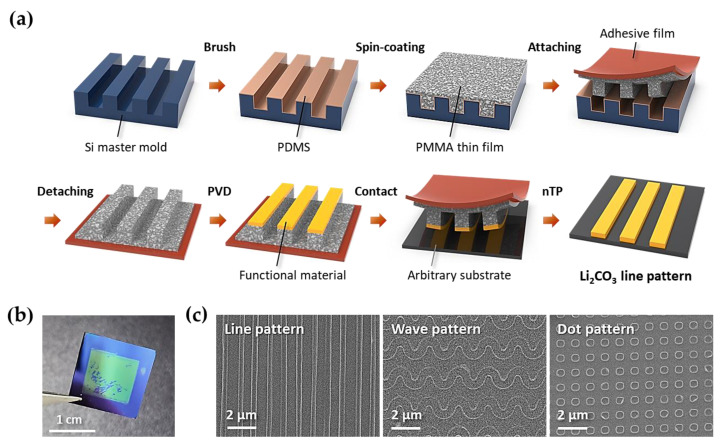
Various Li_2_CO_3_ patterns formed by the nTP process. (**a**) Procedure of the pattern formation of Li_2_CO_3_ nanostructures by nTP. (**b**) Photographic image of a transfer-printed Li_2_CO_3_ pattern on a SiO_2_/Si substrate. (**c**) Various pattern geometries obtained by the nTP process.

By using the Li_2_CO_3_ sputter target, we demonstrate nanoscale pattern formation via the transfer printing method. Figure 3a shows the nTP procedure used to pattern the Li_2_CO_3_ nanostructure. We conducted the nTP process for the Li_2_CO_3_ material on the SiO_2_/Si substrate at chip scale. Figure 3b shows a photographic image of the transfer-printed Li_2_CO_3_ pattern over an area of 1 × 1 cm^2^. Figure 3c shows various Li_2_CO_3_ nanostructures, showing highly ordered line, wave, and dot patterns. These results strongly suggest that many other anode materials of LIBs can be patterned with designable shapes by the nTP process after the fabrication of the sputter target.

**Figure 4:**
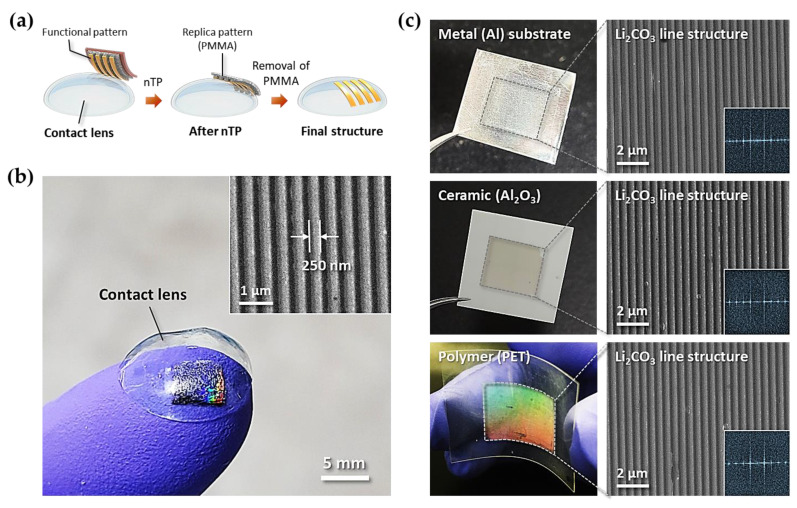
Transfer-printed Li_2_CO_3_ nanopatterns on the various substrates. (**a**) Key process of nTP for the fabrication of Li_2_CO_3_ nanostructures on a contact lens. (**b**) Photographic image of a Li_2_CO_3_ 250-nm-line pattern on the soft contact lens. The inset image is the magnified SEM image of the Li_2_CO_3_ line pattern on the contact lens. (**c**) Transfer-printed line patterns on various material surfaces, in this case metal (Al), ceramic (Al_2_O_3_), and polymer (PET) substrates.

To extend this approach to various devices, we undertook the patterning of functional materials on various surfaces by the nTP process. Figure 4 shows the formation of Li_2_CO_3_ nanopatterns on various surfaces, including a non-planar flexible substrate. A key process during the fabrication of functional patterns on an elastic substrate is schematically illustrated in Figure 4a. As shown in Figure 4b, well-defined Li_2_CO_3_ nanostructures on the surface of a HEMA contact lens were obtained, showing highly ordered line structures with a width of 250 nm. Figure 4c shows transfer-printed Li_2_CO_3_ nanostructures on the various substrates, clearly showing periodic Li_2_CO_3_ line patterns on Al, Al_2_O_3_, and flexible PET over a large area (as shown in the photograph and SEM images in Figure 4c). The insets in the SEM images show fast Fourier transform (FFT) patterns, which indicate the good ordering of the high-resolution line structures. These results imply that the formation of nanopatterns can be applied to most surfaces (planar, curved, elastic, and flexible) of various materials (metal, ceramic, and polymer) by the nTP process.

## 4. Conclusions

In summary, we demonstrated a simple and practical nTP process by which to obtain high-resolution nanostructures of LIB anode materials on the various substrates, such as metal, oxide, and polymer substrates. We effectively fabricated a high-density Li_2_CO_3_ sputter target from a raw powder material by the SPS process. We used the Li_2_CO_3_ sputter target with a RF sputtering system for the generation of high-purity functional nanopatterns. Various Li_2_CO_3_ patterns on the nanoscale were successfully obtained on the SiO_2_/Si substrate by nTP, specifically line, wave, and dot structures. Furthermore, we realized the formation of Li_2_CO_3_ nanoscale patterns on the diverse substrate materials, in this case Al, Al_2_O_3_, flexible PET, and a soft HEMA contact lens, demonstrating the excellent pattern uniformity of the proposed process. We expect that this pattern formation approach, feasible for use with numerous LIB anode materials, will be extended to other emerging research areas related to the structural design of LIB electrodes in the near future.

## Data Availability

The data presented in this study are available on request from the corresponding author.

## References

[1] Sun H., Zhang Y., Zhang J., Sun X., Peng H. (2017). Energy harvesting and storage in 1D devices. Nat. Rev. Mater..

[2] Hu Y., Lin L., Zhang Y., Wang Z.L. (2012). Replacing a battery by a nanogenerator with 20 V output. Adv. Mater..

[3] Seung W., Gupta M.K., Lee K.Y., Shin K.-S., Lee J.-H., Kim T.Y., Kim S., Lin J., Kim J.H., Kim S.-W. (2015). Nanopatterned textile-based wearable triboelectric nanogenerator. ACS Nano.

[4] Abtahi A., Johnson S., Park S.M., Luo X., Liang Z., Mei J., Graham K.R. (2019). Designing π-conjugated polymer blends with improved thermoelectric power factors. J. Mater. Chem. A.

[5] Atanasov V., Lee A.S., Park E.J., Maurya S., Baca E.D., Fujimoto C., Hibbs M., Matanovic I., Kerres J., Kim Y.S. (2020). Synergistically integrated phosphonated poly(pentafluorostyrene) for fuel cells. Nat. Mater..

[6] Miyake J., Ogawa Y., Tanaka T., Ahn J., Oka K., Oyaizu K., Miyatake K. (2020). Rechargeable proton exchange membrane fuel cell containing an intrinsic hydrogen storage polymer. Commun. Chem..

[7] Nayak P.K., Mahesh S., Snaith H.J., Cahen D. (2019). Photovoltaic solar cell technologies: Analysing the state of the art. Nat. Rev. Mater..

[8] Park S.M., Abtahi A., Boehm A.M., Graham K.R. (2020). Surface ligands for methylammonium lead iodide films: Surface coverage, energetics, and photovoltaic performance. ACS Energy Lett..

[9] Hofmann C.L.M., Fischer S., Eriksen E.H., Bläsi B., Reitz C., Yazicioglu D., Howard I.A., Richards B.S., Goldschmidt J.C. (2021). Experimental validation of a modeling framework for upconversion enhancement in 1D-photonic crystals. Nat. Commun..

[10] Palacín M.R. (2009). Recent advances in rechargeable battery materials: A chemist’s perspective. Chem. Soc. Rev..

[11] Jiang Z., Li J., Yang Y., Mu L., Wei C., Yu X., Pianetta P., Zhao K., Cloetens P., Lin F. (2020). Machine-learning-revealed statistics of the particle-carbon/binder detachment in lithium-ion battery cathodes. Nat. Commun..

[12] Trahey L., Brushett F.R., Balsara N.P., Ceder G., Cheng L., Chiang Y.-M., Hahn N.T., Ingram B.J., Minteer S.D., Moore J.S. (2020). Energy storage emerging: A perspective from the Joint Center for Energy Storage Research. Proc. Natl. Acad. Sci. USA.

[13] Chang Z., Li C., Wang Y., Chen B., Fu L., Zhu Y., Zhang L., Wu Y., Huang W. (2016). A lithium ion battery using an aqueous electrolyte solution. Sci. Rep..

[14] Harper G., Sommerville R., Kendrick E., Driscoll L., Slater P., Stolkin R., Walton A., Christensen P., Heidrich O., Lambert S. (2019). Recycling lithium-ion batteries from electric vehicles. Nature.

[15] Ahmadi L., Young S.B., Fowler M., Fraser R.A., Achachlouei M.A. (2017). A cascaded life cycle: Reuse of electric vehicle lithium-ion battery packs in energy storage systems. Int. J. Life Cycle Assess..

[16] Olivetti E.A., Ceder G., Gaustad G.G., Fu X. (2017). Lithium-ion battery supply chain considerations: Analysis of potential bottlenecks in critical metals. Joule.

[17] Hannan M.A., Lipu M.S.H., Hussain A., Mohamed A. (2017). A review of lithium-ion battery state of charge estimation and management system in electric vehicle applications: Challenges and recommendations. Renew. Sustain. Energy Rev..

[18] Li M., Lu J., Chen Z., Amine K. (2018). 30 Years of lithium-ion batteries. Adv. Mater..

[19] Kittner N., Lill F., Kammen D.M. (2017). Energy storage deployment and innovation for the clean energy transition. Nat. Energy.

[20] Xu S., Hessel C.M., Ren H., Yu R., Jin Q., Yang M., Zhao H., Wang D. (2014). α-Fe2O3multi-shelled hollow microspheres for lithium ion battery anodes with superior capacity and charge retention. Energy Environ. Sci..

[21] Zhao Y., Peng L., Ding Y., Yu G. (2014). Amorphous silicon honeycombs as a binder/carbon-free, thin-film Li-ion battery anode. Chem. Commun..

[22] Heiskanen S.K., Kim J., Lucht B.L. (2019). Generation and evolution of the solid electrolyte interphase of lithium-ion batteries. Joule.

[23] Pender J.P., Jha G., Youn D.H., Ziegler J.M., Andoni I., Choi E.J., Heller A., Dunn B.S., Weiss P.S., Penner R.M. (2020). Electrode degradation in lithium-ion batteries. ACS Nano.

[24] Komaba S., Watanabe M., Groult H., Kumagai N. (2008). Alkali carbonate-coated graphite electrode for lithium-ion batteries. Carbon.

[25] Li J., Li M., Guo C., Zhang L. (2018). Recent progress and challenges of micro-/nanostructured transition metal carbonate anodes for lithium ion batteries. Eur. J. Inorg. Chem..

[26] Gao Y., Huang C., Hao C., Sun S., Zhang L., Zhang C., Duan Z., Wang K., Jin Z., Zhang N. (2018). Lead halide perovskite nanostructures for dynamic color display. ACS Nano.

[27] Yao Y., Zhang L., Leydecker T., Samorì P. (2018). Direct photolithography on molecular crystals for high performance organic optoelectronic devices. J. Am. Chem. Soc..

[28] Cox L.M., Martinez A.M., Blevins A.K., Sowan N., Ding Y., Bowman C.N. (2020). Nanoimprint lithography: Emergent materials and methods of actuation. Nano Today.

[29] Chou S.Y., Krauss P.R., Renstrom P.J. (1996). Imprint lithography with 25-nanometer resolution. Science.

[30] Torretti F., Sheil J., Schupp R., Basko M.M., Bayraktar M., Meijer R.A., Witte S., Ubachs W., Hoekstra R., Versolato O.O. (2020). Prominent radiative contributions from multiply-excited states in laser-produced tin plasma for nanolithography. Nat. Commun..

[31] Mojarad N., Gobrecht J., Ekinci Y. (2015). Beyond EUV lithography: A comparative study of efficient photoresists’ performance. Sci. Rep..

[32] Park W.I., Jung Y.K., Kim Y., Shin W.H., Choi Y.J., Park T.W., Shin J.H., Jeong Y.H., Cho J.H., Shin H. (2018). Individual confinement of block copolymer microdomains in nanoscale crossbar templates. Adv. Funct. Mater..

[33] Jung H., Shin W.H., Park T.W., Choi Y.J., Yoon Y.J., Park S.H., Lim J.-H., Kwon J.-D., Lee J.W., Kwon S.-H. (2019). Hierarchical multi-level block copolymer patterns by multiple self-assembly. Nanoscale.

[34] Jung D.S., Bang J., Park T.W., Lee S.H., Jung Y.K., Byun M., Cho Y.R., Kim K.H., Seong G.H., Park W.I. (2019). Pattern formation of metal–oxide hybrid nanostructures via the self-assembly of di-block copolymer blends. Nanoscale.

[35] Onses M.S., Song C., Williamson L., Sutanto E., Ferreira P.M., Alleyne A.G., Nealey P.F., Ahn H., Rogers J.A. (2013). Hierarchical patterns of three-dimensional block-copolymer films formed by electrohydrodynamic jet printing and self-assembly. Nat. Nanotechnol..

[36] Pham T.A., Nguyen T.K., Vadivelu R.K., Dinh T., Qamar A., Yadav S., Yamauchi Y., Rogers J.A., Nguyen N.T., Phan H.P. (2020). A versatile sacrificial layer for transfer printing of wide bandgap materials for implantable and stretchable bioelectronics. Adv. Funct. Mater..

[37] Chanda D., Shigeta K., Gupta S., Cain T., Carlson A., Mihi A., Baca A.J., Bogart G.R., Braun P.V., Rogers J.A. (2011). Large-area flexible 3D optical negative index metamaterial formed by nanotransfer printing. Nat. Nanotechnol..

[38] Cho S.H., Baek K.M., Han H.J., Kim M., Park H., Jung Y.S. (2020). Selective, quantitative, and multiplexed surface-enhanced raman spectroscopy using aptamer-functionalized monolithic plasmonic nanogrids derived from cross-point nano-welding. Adv. Funct. Mater..

[39] Choi M.K., Yang J., Kang K., Kim D.C., Choi C., Park C., Kim S.J., Chae S.I., Kim T.-H., Kim J.H. (2015). Wearable red–green–blue quantum dot light-emitting diode array using high-resolution intaglio transfer printing. Nat. Commun..

[40] Ahn J.-H., Kim H.-S., Lee K.J., Jeon S., Kang S.J., Sun Y., Nuzzo R.G., Rogers J.A. (2006). Heterogeneous three-dimensional electronics by use of printed semiconductor nanomaterials. Science.

[41] Zaumseil J., Meitl M.A., Hsu J.W.P., Acharya B.R., Baldwin K.W., Loo Y.-L., Rogers J.A. (2003). Three-dimensional and multilayer nanostructures formed by nanotransfer printing. Nano Lett..

[42] Meitl M.A., Zhu Z.-T., Kumar V., Lee K.J., Feng X., Huang Y.Y., Adesida I., Nuzzo R.G., Rogers J.A. (2006). Transfer printing by kinetic control of adhesion to an elastomeric stamp. Nat. Mater..

[43] Park T.W., Byun M., Jung H., Lee G.R., Park J.H., Jang H.-I., Lee J.W., Kwon S.H., Hong S., Lee J.-H. (2020). Thermally assisted nanotransfer printing with sub–20-nm resolution and 8-inch wafer scalability. Sci. Adv..

